# Computational Approaches in Detecting Non- Coding RNA

**DOI:** 10.2174/13892029113149990005

**Published:** 2013-09

**Authors:** Chunyu Wang, Leyi Wei, Maozu Guo, Quan Zou

**Affiliations:** 1 School of Computer Science and Technology, Harbin Institute of Technology, Harbin 150001, China;; 2 School of Information Science and Technology, Xiamen University, Xiamen 361005, China

**Keywords:** Non-coding RNA, Machine learning, Bioinformatics, lncRNA, microRNA, Deep sequencing.

## Abstract

The important role of non coding RNAs (ncRNAs) in the cell has made their identification a critical issue in the biological research. However, traditional approaches such as PT-PCR and Northern Blot are costly. With recent progress in bioinformatics and computational prediction technology, the discovery of ncRNAs has become realistically possible. This paper aims to introduce major computational approaches in the identification of ncRNAs, including homologous search, *de novo* prediction and mining in deep sequencing data. Furthermore, related software tools have been compared and reviewed along with a discussion on future improvements.

## INTRODUCTION

In the early biological researches, scientists mainly focused on the prokaryotes, which are dominated (85%-90%) by protein-coding genes [1] and it is publicly considered that the cellular activities are implemented by the proteins which are transcribed from those coding genes. But in the evolution of species, the relative proportions of the coding genes are gradually reducing, whereas the variety of cellular functions increasing. It is estimated that 98% of mammalian genomic output may be non-coding RNAs (ncRNAs), while the remaining 2% encodes the proteins [2]. However, at present, we have incomplete knowledge of those non-coding regions containing both non-coding genes and genomic elements which may regulate gene expression [3]. As a result, we are currently more interested in the non-coding regions which could lead to the better understanding of biological processes. They may be involved in the gene expression control, cancer and aging [61].

As stated in the central dogma of molecular biology, gene sequences (DNA) are transcribed into RNA according to the law of chemistry and physics. Some RNAs, including messenger RNA (mRNA), are called coding RNAs, since they are translated into protein and the others are called non-coding RNAs, because of their function as RNA molecules rather than coding protein. Non-coding RNAs are involved in translation, splicing, gene regulation, chromatin remodeling, gene modification, degradation and other functions [61]. They are also closely associated with cancer and other complex diseases [4]. Many kinds of functional ncRNAs have been discovered with biological experiments and computational methods. In the literature, ncRNAs are divided into several categories [5]. Some ncRNAs are named according to their functions, like microRNAs (miRNA), package RNAs (pRNA) or transfer RNAs (tRNA), etc. Others are named by their cellular localizations, such as piRNAs (interact with piwi protein) and rasiRNAs (repeat associated small interfering RNAs). There are still many other ncRNAs unclassified. 

Non-coding RNAs are recognized only in biological experiments with technologies such as full-length complementary DNA cloning and genomic tiling arrays in the transcriptomes of organisms. Although these technologies can suit long ncRNA (lncRNA) genes in an efficient way, they are costly always requiring enough RNA samples, and are therefore limited. To overcome this shortage, researchers have developed computational biology approaches to discover ncRNAs [6] and are incorporating these computational approaches in experimental methods [7].

Although these computational methods and software tools have their characteristics, a unified framework for identifying all ncRNAs still needs to be discovered due to the diversity of ncRNAs, missing common sequence features and the lack of post-transcriptional processing information. Firstly, there are many kinds of ncRNAs in the species. For example, tRNAs and rRNAs involve in protein production; miRNAs control gene expression; snoRNAs modify post-transcription of other RNA molecules [8]. Different functions are induced by diverse ncRNA structures and there are also variations in ncRNA length. Secondly, they are different from protein-coding genes which have a lot of common conserved features of primary sequence, including splice cites, promoters, terminator and binding motifs etc. There are few common primary features among the non-coding RNAs, so it is difficult to identify all ncRNAs from primary sequence [9]. Therefore, although common primary features may exist in certain ncRNA families, researchers could not simply apply these features for identifying all ncRNAs. Finally, many ncRNAs primarily transcribed from non-coding genes will go through the post-transcriptional processing to reach maturity. The modifications are intended to change their structures, which are closely related to their functions. So far, we are still unable to explain the modifications with the current knowledge and predict them with software programs. Consequently, we try to introduce most currently existing approaches about the identification of ncRNAs.

## METHODS USED IN ncRNA IDENTIFICATION

A lot of computational approaches for detecting ncRNA genes have been designed and reported, but as the variations of ncRNAs, most of these methods are developed for specific ncRNAs or specific ncRNA family. In general, these methods can be divided into two classes.

1. Methods based on homology information. These methods always require homology information and a good quality of alignment among sequences. Only those ncRNAs, which are homologous to known ncRNA family, can be discovered with these methods. Novel computational methods need to be developed for identifying novel ncRNAs.
2. Methods based on common features in ncRNA genes. These methods are called “*de novo*” approaches, which do not require homology information and sequence alignment except known sequence and structural features derived easily from the genome. In addition, the machine learning method known as Random Forest or Support Vector Machine is often used to predict ncRNA genes based on features. In fact, ncRNA gene-finding method based on nucleotide composition like (G+C)% has made some success in some specific species genomes [10]. However, other investigations have indicated that programs based compositions alone are not sufficient to identify ncRNA genes effectively [11]. As a consequence, when using the *de novo* methods for identifying ncRNAs, we can derive features, including sequence and structural features, from sequences and select the appropriate classifier to model these features and train the model to achieve high accuracy of ncRNA prediction [12].

## METHODS FOR HOMOLOGY-BASED ncRNA IDENTIFICATION

It is commonly believed that most ncRNAs are less conserved in sequence [8]. Although there are few common features in ncRNA sequences, it may be different for some special ncRNA families. So common sequence and structural characteristics are used as homology information to detect these ncRNAs. Homology search is to detect all homologous genes in the target sequences, given one or more ncRNA which could represent for a specific ncRNA family. Consequently, we mainly focus on their sequence homology and structure homology, which are based on their features respectively.

There are some software tools based on sequence homology, such as BLAST [13], FASTA [14], S Search [15] and BLAT [16] etc. BLAST (Basic Local Alignment Search Tool) employs a measure of sequence similarities between input sequences and known ncRNAs. A score derived from BLAST approximately quantifies this similarity. However, ncRNAs rarely preserve high degree of the similarity. Furthermore, it relies too heavily on individual sequences rather than focusing on the common features of the ncRNA family. Consequently, a family of homologous sequences is aligned to find the positions conserved than others. And then BLAST is used for finding these common positions in the alignments for target sequence when looking for the additional ncRNA family member. BLAT, which is the abbreviation of “BLAST-like alignment tool”, is similar in many ways to BLAST. When multiple sequences as inputs are aligned to a large sequence database, BLAT performs at higher speed than BLAST. In addition, BLAT has also high sensitivity and specificity for ncRNA detection [12]. In the research of sequence homology, a probabilistic model, named Hidden Markov Model (HMM), which models the features of the homologous sequences, is also used to predict ncRNAs. It builds a model representing the consensus sequence for the family, not the sequence of any particular member [17].

It is naturally hard to identify ncRNAs effectively on sequence level when the level of sequence homology is low. Secondary structure of sequence is more conserved than sequence in the long evolutionary time [18]. Consequently, structure homology is also used to detect ncRNA genes. Programs INFERNAL [18], Rsearch [19] and FastR [20] are all based on structure homology. FastR package is applied to search homologous structure of ncRNAs in large genomic sequence [20]. INFERNAL and Rsearch allow for searching a sequence database for homologous ncRNAs, which are given and structured [15]. Taken tRNA as an example, it is publicly believed that tRNA has a classical “cloverleaf” structure. When Rsearch and BLAST or FASTA are both used to identify tRNA, prediction accuracy from Rsearch is better than that from BLAST or FASTA.

At present, there are many approaches based on a combination of sequence and structure homology to identify ncRNA. For example, when we annotate miRNA genes, known mature miRNAs are mapped into the predicted sequences with BLAST. After that, the mapped sequences are in high level of sequence homology with known miRNA. Then a model is built for the mapped sequences with their structure homology information, including secondary structures, pairwise sequence alignment and structural alignment. Finally, we get a measure of similarity of sequences to annotate true miRNAs, as shown in Fig. (**1**).

Among the current prediction tools based on homology information, most are designed using both sequence homology and structure homology information, such as ERPIN [21] and miRAlign [22]. ERPIN and BLAST are both used to detect new miRNAs. As a consequence, ERPIN increases the number of new miRNA candidates by 17% compared to a BLAST search. The result means that the programs using a combination of sequence and structure homology can get higher accuracy to identify conserved structure ncRNAs than those programs based only on sequence homology. But ERPIN package is limited to identify miRNAs when there are not sufficient known miRNA samples. On the contrary, miRAlign is applicable to identify novel miRNAs with few known miRNA samples. And in order to investigate the ability of miRAlign to identify miRNA in different species, researchers compared miRAlign with BLAST and ERPIN. Consequently, miRAlign achieved higher specificity and sensitivity compared to that exhibited by BLAST and ERPIN searches [18]. As the sequences and structures are deeply researched in miRNA, HMM (Hidden Markov Model) is used for the miRNA precursors [23] and targets [24] identification. For some ncRNAs, when the secondary structure is conserved, such as tRNA and H/ACA box snoRNA, context-sensitive HMM is used for identification [25]. And the non-coding RNA database RFAM is built based on HMM [26].

## METHODS FOR *de novo* ncRNA IDENTIFICATION

We can not discover novel ncRNA families with homology-based methods which rely mostly on homology information. Thus, de novo approaches are developed to solve this problem using features derived from the sequences and structures of known ncRNA genes.

### Methods Based on Sequence Features

In the earlier studies, nucleotide composition was used as sequence features to identify ncRNA in some nucleotide compositional bias species. For example in an AT-rich extreme hyperthermophile, ncRNA genes with a stable secondary structure might be found by calculating GC content**,** which is intended to stabilize their structures in the high temperature environment [8]. However, a single feature is not sufficient to identify ncRNA effectively. As a result, other sequence features have been employed to combine with nucleotide composition to detect ncRNA, including di- and tri-nucleotide frequencies, known RNA motifs and folding energy etc. 

Currently, many programs based on sequence features have been developed, such as CRITICA [27], CST miner [28] and EST scan [29]. Researchers utilize these three programs to identify ncRNA from the 102801 FANTOM sequences respectively and find that CRITICA shows the highest degree of concordance which is up to 94.8% with the other two programs. And its concordance reveals the individual prediction accuracy of each program [30]. Furthermore, the machine learning algorithms are added into ncRNA identification. For example, CONC [31] takes sequence features as input and then uses SVM (Support Vector Machine) to train these features. It has high specificity and sensitivity for ncRNA annotation. However, CONC is slow to the large datasets and spends much computing resources. Compared to CONC, we run CPC [32] on two datasets including one non-coding RNA dataset and one protein-coding dataset respectively and record its result in (Table **1**). What we get from the result is that CPC has higher accuracy and consumes lower time and space than CONC.

### Methods Based on Structure Features

It is publicly known that RNA molecule is a single strand, and usually folds into secondary structure, which is more conserved than primary sequence in long distant evolution. Thus, we investigate approaches for incorporating secondary structure into identification of novel ncRNAs. Actually, the minimum folding energy (MFE) approach is extensively used to predict secondary structure of the target sequences [33]. For example, the programs RNAfold [34], Mfold [35] and Afold [36] all based on this approach have successfully been applied for novel ncRNA identification. To achieve high sensitivity and specificity, an alternative approach, Sfold also incorporates a probabilistic model in the prediction [37]. In addition, searching novel H/ACA snoRNA in the yeast or other eukaryote genomes, the approach based on MFE structure could also provide good prediction [38].

However, secondary structure alone is generally not efficient enough for the detection of ncRNAs [39]. For a given sequence, it might fold into different secondary structures but these structures intend to have similar MFE. Thus, other structure features are extensively discovered and applied to distinguish ncRNA from the target sequences, such as thermodynamic stability and shannon entropy etc. For example, in a data set, containing real ncRNAs and their di-shuffled sequences, the di-shuffled sequences are intended to have higher MFE and Shannon entropy than the real ncRNAs [40]. In addition, a program MiPred [41], employs a combination of features, containing local contiguous structure-sequence composition, MFE and P-value of randomization test, uses a novel machine-learning technique based on random forest algorithm to identify putative miRNA precursors and seems to provide high sensitivity and specificity. Furthermore, PlantMiRNAPred [42] can classify plant miRNA precursors efficiently by SVM together with feature and sample selection strategies. It selects a variety of features from both primary sequence and secondary structure and provides a viable method for discovering novel plant pre-miRNAs. However, all these methods and software mentioned above suit for the long DNA sequences, such as EST or genome data. When dealing with deep sequencing or the next generation sequencing data, it needs more mapping or assembling strategies.

### Methods Based on Deep Sequencing Technology

With the development of the next generation sequencing technologies, it has been implemented for small ncRNAs discovery, particularly for miRNAs. Massively next generation sequencing technologies (also named deep sequencing) are currently in widespread use, including 454, Solexa and SOLiD. Compared to conventional sequencing technologies, deep sequencing technologies accelerate biological research and significantly reduce the cost. Here we present currently available tools for miRNA identification with the deep sequencing technologies, including miRDeep [43], CID-miRNA [44], MiRank [45], miRCat (identify plant miRNAs) [46], mirTool [47], and miRanalyzer [48].

MiRNA discovery with deep technologies is generally divided into two steps. The first step is called filtering. The sequence reads derived from deep sequencing are mapped to the whole genome. The reads that map to tRNA or sRNA, etc are discarded and then the remaining reads are mapped to known miRNA database again. The sequence reads that map to the known miRNA database are passed and recognized as miRNA candidates. The other step is called modeling. The miRNA candidates are simply modeled by some algorithms. For example, in the core algorithm of miRDeep, potential miRNA candidates are modeled for the combined compatibility of energetic stability, positions and frequencies of reads with Dicer processing [33]. A number of features contribute to the final score derived from the model. miRDeep could discover not only known and novel miRNAs but also provide a statistical evaluation of false positive rate and sensitivity, which most machine learning algorithm could not provide. The flow is shown in Fig. (**2**).

However, compared to other tools, miRDeep relies on the characteristic pattern of high expression, thus it is limited for the miRNA at low level of expression. In this situation, it needs researchers to explore other means to indentify novel miRNA in the low expression sequences. For example, the conservation pattern of structure can be used to discover miRNA precursors [49]. Firstly, taking the sequence reads to map the whole genome, we can remove those reads that do not map to genome then fold remaining reads with RNA by Vienna package [25]. The novel hairpins produced by Vienna are filtered, while those single –loop hairpins with mature-miRNA in one side of hairpin are passed as possible hairpins. Secondly, these possible hairpins are refolded by the Vienna package and filtered again with Ambros criteria [50]. Finally, real mature-miRNA can be discovered in the remaining hairpins. 

MiRanalyzer, which is similar to miRDeep, could search known miRNA in the miRNA database and discover novel miRNA, particularly those undiscovered miRNA family. The core algorithm of MiRanalyzer is a sensitive machine learning method using random forest algorithm. And the feature selection technologies are also used in the MiRanalyzer. Subsequently, the prediction of new miRNAs using MiRanalyzer could reach high sensitivity and keep a low level of false positive rate [38]. Similar to miRDeep and MiRanalyzer, MirTool also can predict known and novel miRNAs. Furthermore, it could provide detailed information for the known miRNAs, such as miRNA/miRNA* and absolute/relative reads count [37]. Here is another program called miRank using random walk-based ranking algorithm. The miRank method has some remarking properties. For example, it does not rely on cross-species conservation so that it can identify species-specific miRNAs. In addition, it does not require a number of miRNA samples but could reach a high discover accuracy. Hence, miRank is a useful tool for the miRNA identification [35]. Besides using deep sequencing technologies to detect miRNAs, other small ncRNA molecules such as snoRNA, piRNA, endo-siRNA, are also identified by deep sequencing technologies. For example, we can apply SnoSeeker which can identify snoRNAs from deep sequencing data [51]. (Table **2**) lists the main software tools for the ncRNA discovery.

Indeed, the main disadvantage of the popular software tools is that they are designed specially for just one kind of ncRNAs. For example, tRNAscanSE is used for detecting tRNA; snoSeeker is to look for snoRNA; miRDeep, miRCat, mirTool, miRanalyzer and MIReNA are all designed for mining microRNA. So there are working repeat and confusion for the conflict result when we annotate new sequencing data. Moreover, they are unfair to compare for ncRNA annotation.

Two methods, i.e., CSHMM and MIReNA, are employed for comparison. CSHMM is the machine learning based method used either to analyze individual sequences or scan potential pre-miRNAs from human genome-scale data. MIReNA is the method based on a genome-wide search algorithm for pre-miRNAs search. Both methods are used to search for known pre-miRNAs from the Chr 19. Results show that 70 and 74 true positives are correctly predicted by CSHMM, and MIReNA, respectively. Regarding methods to detect pre-miRNAs from human genome-scale data, maintaining high specificity is even of greater importance. MIReNA correctly predicts the highest number of true positives, but also produces 10,626 pre-miRNA candidates, while CSHMM predicts 18,258 false true results. This large number of pre-miRNA candidates is perhaps due to the low specificity of MIReNA. Similarly, the low specificity results in CSHMM as a poor choice for the identification of pre-miRNAs. Actually, MIReNA is capable of performing in different modes when handling different data types (e.g., the genome data, and the deep-sequencing data). It is believed that MIReNA can achieve better performance when evaluated on a more comprehensive data.

In the general, *de novo* methods use machine learning algorithms to train the features from sequence, structure and deep sequencing data to identify ncRNAs. With the development of bioinformatics, more and more features are derived and used in the ncRNA discovery. However, in most cases, these features are redundant. In order to reduce the redundancy, features selection technologies are created and applied to *de novo* methods. Now the main feature selection technologies constantly used are Filter, Wrapper and Embedded technologies [52]. On the other hand, different machine learning methods employed in the discovery are intended to lead to different efficiency and accuracy. Compared to those classifiers, such as SVM and Bayesian, an integrated machine learning model called incRNA is developed and can significantly improve the results in the ncRNA identification [53].

## lncRNA AND lncRNA IDENTIFICATION

Besides small non-coding RNAs, there are long non-coding RNAs (lncRNA), which are longer than 200nt. They can be categorized into long intronic non-coding RNA and intergenic non-coding RNA. They are considered to regulate gene expression through changes in chromatin state, implicate in cancer pathogenesis and correlate with clinical features [58]. With the increasing amount of lncRNAs, identification and function research for lncRNA is called lncRNome [59].

Since the lack of conservation among lncRNA primary sequences, detecting lncRNAs from genomes relies on expression analysis that makes comprehensive characterization of lncRNome difficult. The latest GENCODE has specially collected lncRNAs. First, the transcriptome data were annotated and the protein coding sequences were filtered. Then short sequences, which were shorter than 200nt were removed and the remaining ones were viewed as lncRNAs [56]. Since the lack of experimental transcriptome data, computational prediction of lncRNAs is necessary and meaningful. Machine learning method based on SVM was employed for detecting polycomb-associated lncRNAs [57]. It can distinguish lncRNAs from transcription noise. However, features extraction for lncRNA is a challenging task since lncRNAs are largely unstructured. So regulation elements such as enhancers or promoters are always utilized. Although lncRNAs do not have common secondary structures, structure features can be used for distinguishing lncRNAs with other small ncRNA precursors [60].

## DISCUSSIONS

Although many ncRNA families have been discovered by variety of identification tools, there is currently no unified prediction tool which could detect all kinds of ncRNA. For the specific ncRNA research, it will lead us to develop different programs. In fact, most current approaches and tools are complementary. In order to improve specificity and sensitivity as well as reduce false-positive, we are interested at how to combine these methods and tools effectively. This process seems a little complicated, because it requires us to evaluate different combinational methods, which represent another direction of ncRNA identification.

In the field of ncRNA identification based on homology information, the selection of window size of alignment sequences would be a problem to limit us to use sequence homology methods, because the fixed alignment programs typically assume a window size to reduce computational requirements. In addition, the window size not suitable for the sequence alignments might reduce the prediction accuracy. When level of sequence homology is relatively low, alternative methods based on structure homology are applicable to detect new ncRNAs. Structural alignment approaches are incorporated into the structure homology research. And how to improve speed of structural alignments and their accuracy becomes another area of active research. 

In *de novo* methods, most of them are based on the features derived from sequence and structure. By utilizing these features, kinds of classifiers have been applied to the research. At present, how to combine these features and select a proper classifier represent another direction in the field of ncRNA identification. With the quick development of next generation sequencing technologies, massive sequencing data provides a great deal of power to the ncRNA research.

Computation detecting methods mentioned above are mostly designed for the short non-coding RNAs, such as miRNAs, tRNAs, siRNAs, piRNAs, etc. When dealing with long non-coding RNAs (lncRNA), the computation methods always can not work well. To our knowledge, RT-PCR or CHIP-SEQ is the main detecting method for lncRNA [54, 55]. More research ought to be done on the lncRNA and the computational detecting methods are required for decreasing the molecular biology experiments cost.

In conclusion, ncRNA research is still at its infancy. To get more knowledge about complex ncRNA world, we still have to explore other novel methods, either biological experiments or computational methods. However, since experimental technology has not yet been developed, we should still focus on exploring ncRNA world by computational tools. And novel insights do not only help us to increase our knowledge about RNA world, but also help us to improve computational tools for further identification. Besides detection methods, more computation methods and tools need to be researched deeply, including identification alternative splicing or SNP in ncRNA. They are both interesting and important for the function research of ncRNA.

## Figures and Tables

**Fig. (1):**
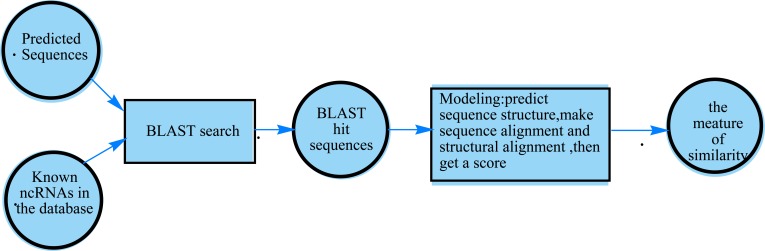
Overview of approaches based on a combination of sequence and structure homology.

**Fig. (2):**
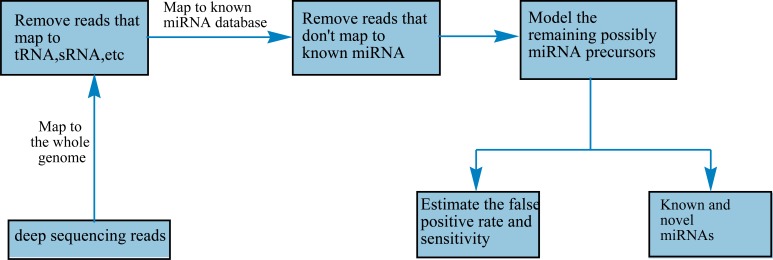
Flow chart of miRNA discovery using deep sequencing technology.

**Table 1.** Evaluation of Accuracy and CPU Time of CPC and CONC on Two Datasets

Dataset	Dataset type	Dataset size	Accuracy		Time(min)	
			CPC	CONC	CPC	CONC
Rfam	Non-coding	9020	87.57%	85.36%	1053	13594
Embl cds	Coding	8949	93.24%	92.93%	5073	60647

**Table 2.** The Main Software Tools of ncRNA Discovery

Homology-based ncRNA identification methods	BLAST, Blat, INFERNAL, FASTA, SSEARCH, Rsearch, FastR, ERPIN, miRAlign	
De novo-based ncRNA identification methods	Sequence features-based methods	CRITICA, CSTminer, ESTscan, CONC, CPC
	Structure features-based methods	RNAfold, Mfold, Afold, MiPred
	Deep sequencing-based methods	miRDeep, CID-miRNA, MiRank, miRCat, mirTool, snoSeeker, MiRanalyzer
